# Percutaneous Thrombin Injection for Treatment of a Hepatic Arterial Pseudoaneurysm after the Placement of a Transjugular Intrahepatic Portosystemic Shunt

**DOI:** 10.25259/JCIS_87_18

**Published:** 2019-05-24

**Authors:** Daniel C. Oppenheimer, Luann Jones, Ashwani Sharma

**Affiliations:** Department of Imaging Sciences, University of Rochester Medical Center, Rochester, NY, USA.

**Keywords:** Hepatic artery pseudoaneurysm, Portal hypertension, Transjugular intrahepatic portosystemic shunt

## Abstract

Transjugular intrahepatic portosystemic shunt (TIPS) is a widely accepted option for treating the complications of portal hypertension. The procedure involves creating a communication between the portal and hepatic venous systems using imaging guidance, thereby diverting the portal venous flow and reducing the portosystemic gradient. However, as with any procedure, TIPS insertion is not without potential complications. We present a case of a 37-year-old female who developed a hepatic artery pseudoaneurysm following the placement of a TIPS which was successfully treated with percutaneous thrombin injection.

## INTRODUCTION

Transjugular intrahepatic portosystemic shunt (TIPS) is a procedure to treat complications of portal hypertension, such as variceal bleeding and recurrent ascites. First described in 1969,^[1]^ long-term patency rates for TIPS have dramatically improved in the last decade with the advent of polytetrafluoroethylene-covered stents.^[2]^ The TIPS redirects portal venous flow to the hepatic veins, bypassing the liver parenchyma, and thereby reducing the portosystemic gradient. A variety of vascular and nonvascular complications have been described following TIPS placement. Hepatic artery pseudoaneurysm is a rare complication, and to our knowledge, this is the first described case of a hepatic artery pseudoaneurysm following TIPS placement which was successfully treated with percutaneous thrombin injection.

## CASE REPORT

A 37-year-old patient with a past medical history of alcoholic cirrhosis, portal hypertension, and refractory ascites was admitted for elective TIPS placement. The patient underwent TIPS placement which reduced the portosystemic gradient from 13 mmHg to 7 mmHg. After a short period of standard postprocedural observation, the patient was discharged home with plans for routine follow-up TIPS ultrasound (US) in 1 week.

**Figure 1:**
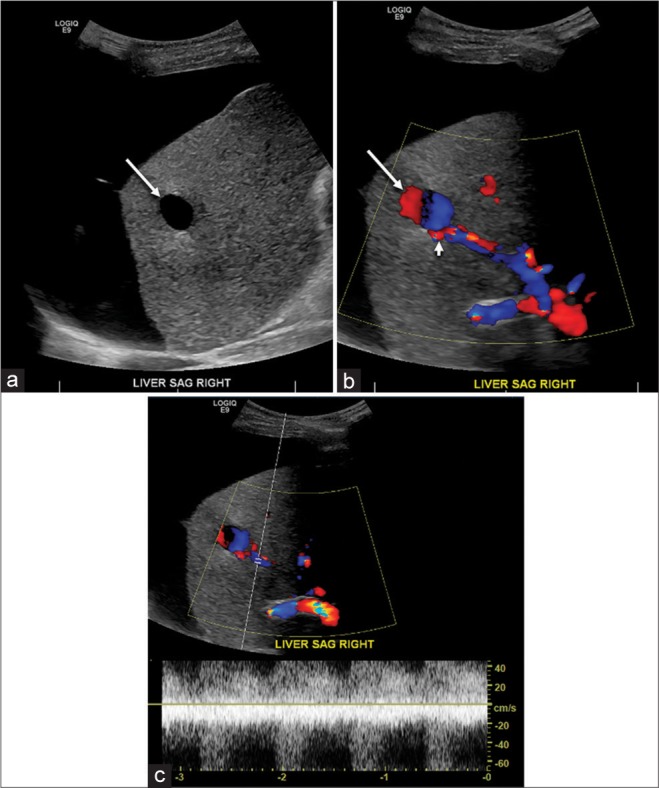
(a) Sagittal gray-scale image of the liver demonstrates a 1.5-cm anechoic lesion in the right hepatic lobe (arrow) with minimal through transmission. Also note moderate volume simple perihepatic ascites. (b) Sagittal color Doppler image of the liver shows the anechoic lesion in the right hepatic lobe with yin-yang pattern of internal flow (arrows) and a vascularized neck (arrowhead).(c) Sagittal color Doppler image of the right hepatic lobe lesion demonstrates a high-velocity low-resistance spectral waveform in the neck of the lesion.

Follow-up US showed a patent TIPS with normal TIPS velocities and patent hepatic vasculature with expected hepatofugal flow in the right and left portal veins. In addition, a 1.5-cm round anechoic lesion was detected in the right hepatic lobe with a “yin-yang” pattern of flow on color Doppler imaging as well as a neck with high-velocity and low-resistance flow [Figure 1]. The US also showed simple appearing perihepatic ascites without any complex components to suggest hemorrhage. The patient’s vital signs were normal and laboratories did not show anemia.

**Figure 2:**
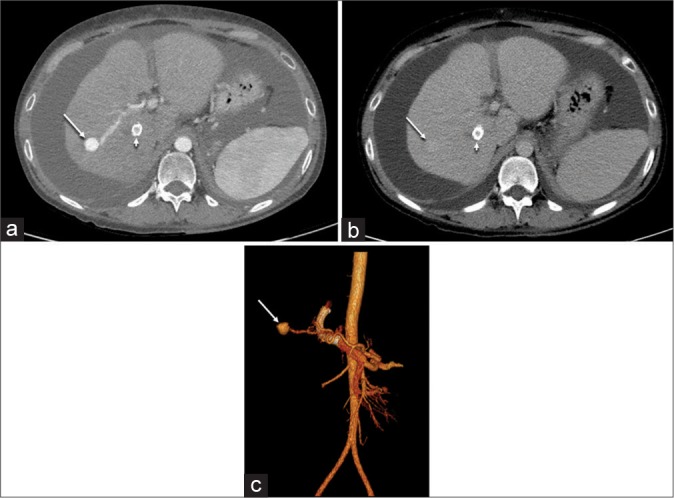
(a) Axial computed tomography angiography in the arterial phase demonstrate a round 1.5-cm lesion in the right hepatic lobe (arrow) with contrast enhancement that matches the arterial blood pool. The newly placed transjugular intrahepatic portosystemic shunt is also visible (arrowhead). (b) Axial contrast-enhanced computed tomography image in the delayed phase demonstrates a faintly enhancing lesion in the right hepatic lobe (arrow) with enhancement that matches the blood pool. The newly placed transjugular intrahepatic portosystemic shunt is also visible (arrowhead). (c) Volume-rendered image from computed tomography angiography demonstrates the hepatic artery pseudoaneurysm (arrow).

Computed tomography angiography (CTA) was subsequently performed after the administration of 150 cc Omnipaque 350 (Iohexol, GE Healthcare Inc., Marlborough, MA, USA) which showed a moderate volume of simple fluid attenuation abdominal ascites and a round 1.5-cm enhancing lesion in the right hepatic lobe corresponding to the finding on US. The lesion showed contrast enhancement which matched the blood pool on the arterial phase and delayed images [Figure 2]. The combined CT and US findings were diagnostic of hepatic artery pseudoaneurysm.

**Figure 3:**
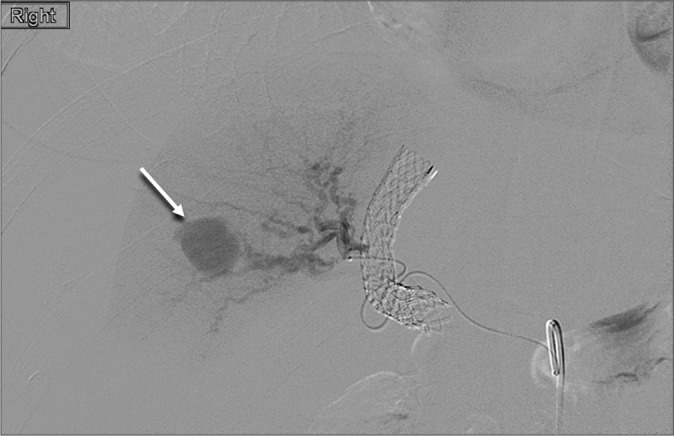
Conventional angiography with selective catheterization of the right hepatic artery demonstrates the enhancing pseudoaneurysm (arrow).

The patient was then referred to interventional radiology for the evaluation and treatment of the hepatic artery pseudoaneurysm. Conventional angiography was performed using a Renegade Microcatheter (Boston Scientific, Marlborough, MA, USA) confirming the pseudoaneurysm arising from a branch of the right hepatic artery [Figure 3]. However, due to the tortuosity of the hepatic artery in this cirrhotic patient, it was not possible to position the microcatheter directly at the aneurysm neck. Transcatheter embolization more proximally would have resulted in nontarget embolization and potentially hepatic ischemia.

**Figure 4:**
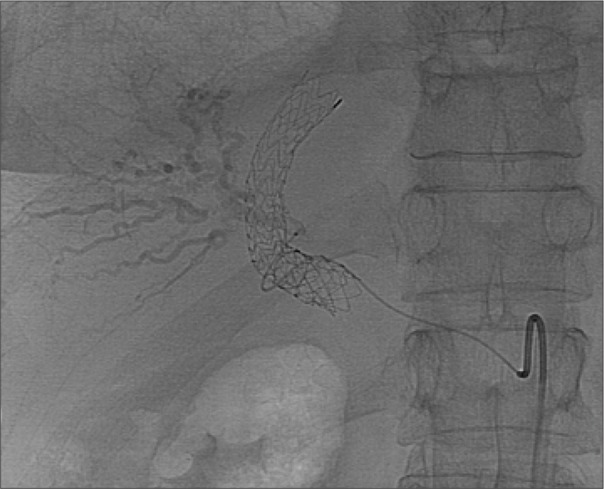
Conventional angiography with selective catheterization of the right hepatic artery following percutaneous thrombin injection demonstrates nonopacification of the pseudoaneurysm compatible with complete thrombosis.

**Figure 5:**
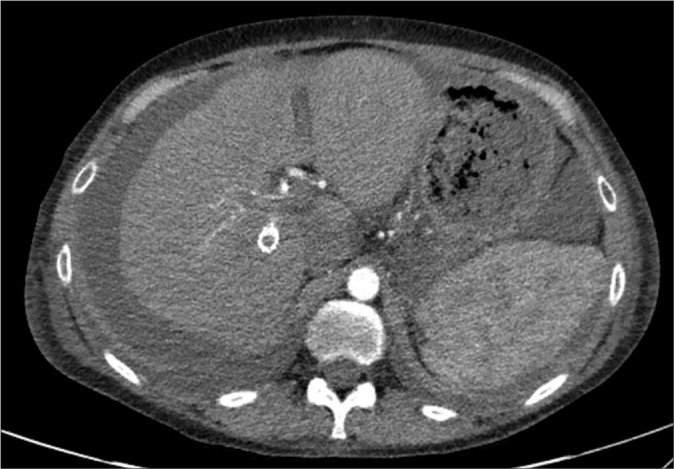
Axial computed tomography angiography performed the day following thrombin injection confirms complete thrombosis of the pseudoaneurysm which is no longer visible.

Therefore, a decision was made to occlude the pseudoaneurysm percutaneously with thrombin injection using US guidance. Thrombin (Recothrom, Mallinckrodt Pharmaceuticals, Hazelwood, MO, USA) was reconstituted and diluted to a concentration of 100 U/ml. Then, using a 5 MHz US transducer (GE Healthcare Inc., Wauwatosa, WI, USA), the pseudoaneurysm was accessed percutaneously with a 25-gauge needle and the needle was directed away from the neck. About 100 U of thrombin was slowly injected into the pseudoaneurysm with continuous US visualization. Color and spectral Doppler images demonstrated immediate cessation of flow in the pseudoaneurysm. Postthrombin injection conventional angiography showed no visible flow in the aneurysm [Figure 4] and preserved arterial flow to the surrounding hepatic parenchyma. Repeat CTA was performed the following day which confirmed a lack of contrast enhancement within the pseudoaneurysm [Figure 5]. There were no surrounding hepatic parenchymal changes to suggest any associated ischemic insult. The patient was discharged home and has done well clinically in the subsequent months.

## DISCUSSION

The hepatic artery and portal vein are situated in close proximity within the portal triad, and therefore, hepatic arterial branches may be inadvertently transgressed during TIPS placement. Potential complications of hepatic arterial puncture include bleeding, thrombosis, arteriovenous fistula, and pseudoaneurysm. While most arterial punctures during TIPS do not result in clinical sequelae, serious complications including death have been reported from hepatic artery injury during TIPS placement.^[3]^

Small hematomas following TIPS are common and are typically followed with serial imaging and treated conservatively unless the hematoma increases in size. Hepatic arterial catheterization is rare during TIPS (1.2%)^[4]^ but would presumably increase the risk of developing a pseudoaneurysm. As with pseudoaneurysms elsewhere in the body, hepatic artery pseudoaneurysms may enlarge or rupture into the adjacent structures (portal vein, hepatic vein, or bile ducts), extend beneath the hepatic capsule (subcapsular hematoma) or through the capsule into the peritoneum (hemoperitoneum).^[5]^

If the arterial injury is recognized at the time of TIPS placement, some authors advocate immediate catheter-directed embolization of the transparenchymal tract leading to the hepatic artery to prevent further bleeding and pseudoaneurysm formation.^[2]^ However, transcatheter hepatic artery embolization may cause hepatic ischemia, which can result in hepatic insufficiency. The risk of hepatic ischemia may be increased in the setting portal hypertension because the portal flow is reduced and these patients are more dependent on hepatic arterial inflow to perfuse the liver. In addition, hepatic arteries often become tortuous in the setting of cirrhosis, and accessing the pseudoaneurysm neck through an endovascular approach may challenging if not be possible, as in our patient’s case. Therefore, percutaneous thrombin injection may be a more suitable treatment option to treat pseudoaneurysms in cirrhotic patients. Percutaneous thrombin injection can also be repeated if the pseudoaneurysm has recannulized on the follow-up US. In addition, compared with transcatheter embolization, thrombin injection is generally a less time-consuming procedure, does not require arteriotomy or sedation, and does not expose the patient to additional ionizing radiation. The recurrence rate of hepatic arterial pseudoaneurysms is not known, but as with femoral artery pseudoaneurysms, recurrence is probably more common with pseudoaneurysms with wide necks.

## CONCLUSION

Hepatic artery pseudoaneurysm is a rare complication following TIPS. The appearance on US, CT and conventional angiography is characteristic. While other authors have described their experience treating TIPS-associated hepatic artery pseudoaneurysms with transcatheter embolization, we report a case which was treated successfully with percutaneous thrombin injection.
